# Mediastinal Hematoma and Tracheal Compression following Transradial Percutaneous Coronary Intervention

**DOI:** 10.1155/2018/6790120

**Published:** 2018-02-22

**Authors:** Nathaniel R. Smilowitz, Muhamed Saric, Michael J. Attubato, James N. Slater

**Affiliations:** Division of Cardiology, Department of Medicine, NYU School of Medicine, NYU Langone Health, New York, NY, USA

## Abstract

Vascular complications from transradial percutaneous coronary intervention (PCI) are rare. We report an unusual case of stridor after PCI due to brachiocephalic artery perforation, pseudoaneurysm formation, and development of a large mediastinal hematoma with tracheal compression. Endovascular repair of the brachiocephalic artery was achieved with covered stent placement at the neck of the pseudoaneurysm. This case highlights the importance of careful guide catheter placement from the right radial approach. Ultimately, rapid diagnosis of vascular perforation, appropriate airway management, and prompt endovascular repair of the injured vessel is critical to the successful management of this life-threatening condition.

## 1. Introduction

Percutaneous coronary intervention (PCI) via the radial artery is an effective approach to treat patients with coronary artery disease. In large randomized trials of transradial versus transfemoral arterial access, patients who underwent transradial catheterization had fewer major adverse cardiovascular events, fewer access site bleeding events, and lower mortality than those who underwent transfemoral coronary intervention [1–3]. Thus, transradial access has been hailed as the preferred approach to minimize vascular complications associated with cardiac catheterization. Major vascular complications of transradial arterial access, although rare, occur in approximately 0.2% of coronary procedures [4, 5]. Vascular perforation, one of the most severe complications of radial PCI, can occur following aggressive manipulation of guidewires or catheters as they are advanced through the radial, brachial, subclavian, and brachiocephalic arteries. Complications typically occur when wires or catheters shear the arterial wall in looped, tortuous, or small caliber arterial segments. In this report, we present an unusual case of stridor after PCI due to brachiocephalic artery perforation, pseudoaneurysm formation, and development of a large mediastinal hematoma with tracheal compression.

## 2. Case Report

A 69-year-old man with a history of hypertension, dyslipidemia, and type 2 diabetes mellitus presented with lifestyle-limiting angina. He underwent diagnostic coronary angiography that revealed stenoses of the left anterior descending (LAD) coronary artery, ramus intermedius coronary artery, and the distal right coronary artery (RCA). Coronary artery bypass grafting was recommended, but the patient refused surgery and elected for percutaneous coronary intervention (PCI) instead. Initially, transradial PCI of the LAD and ramus coronary arteries was performed with placement of drug-eluting stents in each vessel. The patient was discharged on aspirin and ticagrelor with a plan for staged coronary intervention of the distal RCA at a later date.

**Figure 1:**
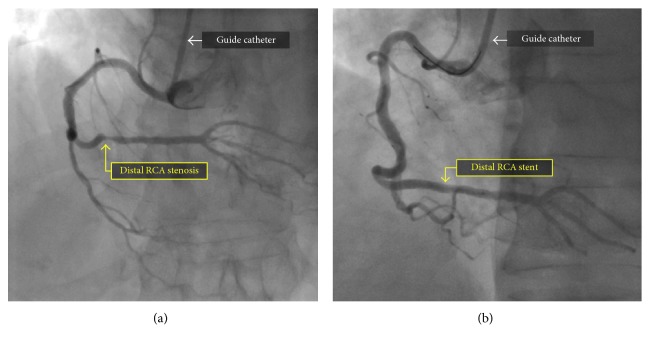
Coronary angiography of the right coronary artery in the left anterior oblique view before (a) and after (b) percutaneous coronary intervention and drug-eluting stent placement.

**Figure 2:**
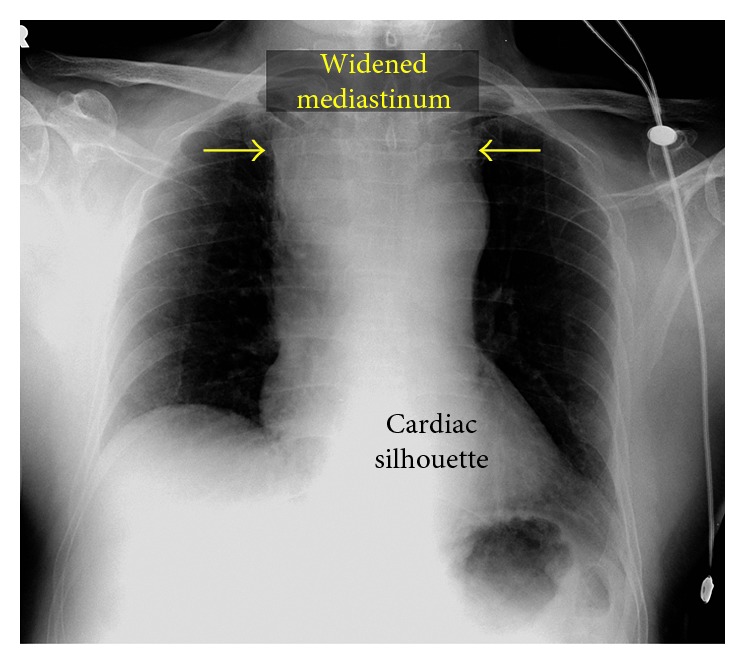
Chest radiography (AP orientation) demonstrating a widened superior mediastinum.

Three months after the initial coronary intervention, the patient returned for planned PCI of the distal RCA stenosis. Access was again obtained in the right radial artery with a short 6 French slender sheath. After routine diagnostic coronary angiography confirmed a severe stenosis in the distal RCA (Figure 1(a)), bivalirudin was administered. A 6 French AL 0.75 guide catheter (Medtronic, Minneapolis, MN, USA) was advanced over an exchange length 0.035″ J-wire, but could not be delivered to the ascending aorta due to resistance in the brachiocephalic artery. The guide was removed and exchanged for a 6 French JR 4 guide catheter (Medtronic, Minneapolis, MN, USA), which was easily advanced into the ascending aorta and used to engage the ostium of the RCA without complication. Next, a standard 0.014″ RunThrough coronary guidewire (Terumo, Tokyo, Japan) was placed in the distal RCA, and percutaneous coronary intervention was performed. The lesion was predilated with a 2.0 mm Maverick compliant balloon (Boston Scientific, Marlborough, MA, USA) inflated to 10 ATM, and a Xience Alpine 2.75 × 12 mm cobalt chromium everolimus drug-eluting stent (Abbot Vascular, Abbott Park, IL, USA) was delivered to the lesion and deployed at 16 ATM. There was a good angiographic result of the distal RCA with no residual stenosis (Figure 1(b)). Immediately following completion of the procedure, the patient reported an episode of chest discomfort, a new cough, bilateral expiratory wheezes, and respiratory stridor. A transient episode of hypotension was noted but resolved without intervention. The patient was rapidly administered IV methylprednisolone, famotidine, diphenhydramine, and inhaled racemic epinephrine for presumed allergic reaction to iodinated contrast. Although his symptoms largely improved, stridor persisted. Urgent otolaryngology consultation was obtained for the evaluation of possible laryngeal edema, but flexible fiber-optic laryngoscopy was unremarkable. Next, chest radiography was promptly obtained, which revealed a markedly widened superior mediastinum (Figure 2).

**Figure 3:**
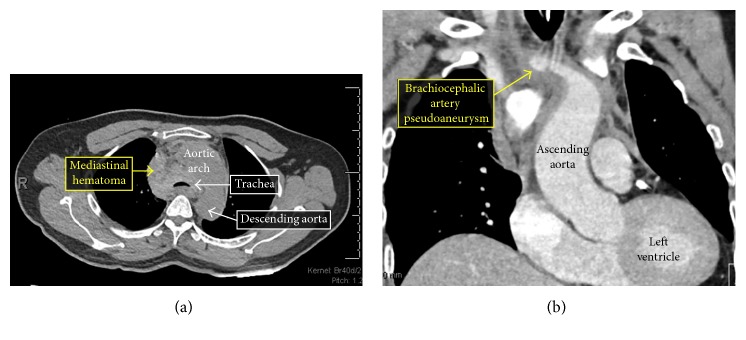
Computed tomography (CT) of the chest without intravenous contrast demonstrating a hyperdense region surrounding the trachea suggestive of an anterior mediastinal hematoma (a). Computed tomography (CT) of the chest with intravenous contrast demonstrating a small pseudoaneurysm arising from the inferior aspect of the brachiocephalic artery (b).

Based on the findings on chest radiography, transesophageal echocardiography (TEE) was urgently performed. Echocardiography revealed no evidence of aortic dissection, but vague echodensities were noted anterior to the right heart suggestive of a hematoma. Thus, computed tomography (CT) of the chest without intravenous contrast was performed to obtain cross-sectional imaging of the mediastinum. The chest CT revealed a large hyperdense region surrounding the trachea suggestive of an anterior mediastinal hematoma, resulting in severe airway compression (Figure 3(a)). Due to the severity of tracheal compression, the decision was made to perform endotracheal intubation and mechanical ventilation and transfer to the intensive care unit. After intubation, a repeat CT of the chest with administration of intravenous contrast was notable for a small pseudoaneurysm arising from the inferior aspect of the brachiocephalic artery, as well as significant narrowing of the distal trachea below the endotracheal tube (Figure 3(b)).

**Figure 4:**
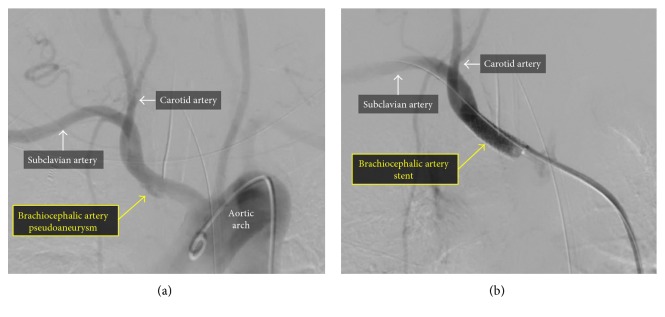
Angiography of the brachiocephalic artery demonstrating a small pseudoaneurysm (a). There was an excellent angiographic result after deployment of a covered stent (b).

After consultation with cardiothoracic and vascular surgery, the decision was made to pursue endovascular repair of the brachiocephalic artery pseudoaneurysm and presumed site of vascular perforation. Access was obtained in the left common femoral artery, and angiography of the brachiocephalic artery was performed, which confirmed the presence of a brachiocephalic artery pseudoaneurysm (Figure 4(a)). Next, a SupraCore wire (Abbot Vascular, Abbott Park, IL, USA) was placed in the right subclavian artery and a 10 × 40 mm iCAST covered stent (Atrium Medical, Hudson, NH, USA) was deployed across the neck of the pseudoaneurysm. The covered stent was postdilated with a 12 × 20 mm Armada balloon (Abbot Vascular, Abbott Park, IL, USA). Following the intervention, there was an excellent angiographic result with preserved subclavian and carotid runoff (Figure 4(b)). Aspirin was continued; P2Y12 inhibitors were temporarily withheld.

Due to persistent tracheal compression after endovascular repair of the brachiocephalic artery, a Tracheobronxane Dumon silicone tracheal stent (Novatech, La Ciotat, France) was inserted by interventional pulmonology to maintain airway patency while the mediastinal hematoma resorbed. Over the ensuring days, serial imaging demonstrated no further enlargement of the mediastinal hematoma, and dual antiplatelet agents were resumed. The tracheal stent was retrieved and removed after 12 days. The patient was ultimately discharged to inpatient rehabilitation on hospital day 19.

## 3. Discussion

Vascular perforation associated with delivery of a 6 French guide catheter in the subclavian and brachiocephalic arteries is rare, with few cases reported in the literature [6–8]. In this case, brachiocephalic artery perforation resulted in pseudoaneurysm formation and a large mediastinal hematoma with tracheal compression and stridor. This highlights the importance of careful guide catheter placement from the right radial approach. When guide catheters are advanced through tortuous subclavian and brachiocephalic arteries, the application of forward pressure against resistance can lead to vascular complications, even when a guidewire is appropriately positioned in the ascending aorta. If resistance is encountered, fluoroscopy should be used to confirm the relative positions of the distal guidewire and guide catheter, and care should be taken to ensure appropriate guidewire position before advancing the guide further. Abduction of the arm and deep inspiration may also facilitate advancement of guide catheters to the ascending aorta. When the guide catheter cannot be easily advanced, balloon-assisted tracking (or similar techniques employing a low-profile 0.035″ compatible support catheter protruding through the lumen of a standard 6 Fr guiding catheter) can smooth the transition at the distal tip of the guide catheter. This can prevent vessel shearing from the “razor effect” at the distal tip of the guide catheter and minimize vascular trauma. Hydrophilic guiding catheters may also be used to pass tortuous subclavian or brachiocephalic anatomy. Rigid guide catheters, larger French sizes, and those with a more tortuous or curved shape at the distal tip may be most likely to cause trauma during catheter delivery. In the present case, the Amplatz shape of the initial guiding catheter may have played a role in the resulting perforation.

This report also illustrates the importance of maintaining a broad differential diagnosis when respiratory distress occurs after PCI. In this case, an allergic reaction to iodinated contrast was initially suspected when stridor and wheezing developed after completion of the procedure. Reactions to contrast media affect approximately 1 in every 500 patients undergoing coronary angiography in the cardiac catheterization laboratory, and the clinical presentation can range from urticaria to laryngeal edema and bronchospasm to cardiovascular shock and respiratory arrest [9]. The likelihood of an anaphylactoid reaction to contrast media is not necessarily related to contrast media dose, nor is it always an immediate response to contrast media exposure. When angioedema is suspected, patients require prompt airway evaluation and may require tracheal intubation to maintain airway patency. In this case, flexible fiber-optic laryngoscopy was urgently performed to exclude this dangerous diagnosis. Once it was evident that laryngeal edema was absent and anaphylactoid reaction to contrast media was unlikely, alternate diagnoses were considered. Ultimately, this rare vascular complication of PCI was only identified after a series of diagnostic imaging studies, including chest radiography, transesophageal echocardiography, computed tomography, and finally, invasive angiography. Therefore, all providers in the cardiac catheterization laboratory should be aware of procedural risks specific to the transradial approach.

In conclusion, vascular perforations due to guide catheter advancement from the right radial artery are rare. In this case, a 6 French AL 0.75 guide catheter led to brachiocephalic artery perforation, pseudoaneurysm formation, and a large mediastinal hematoma with tracheal compression. Prompt recognition, appropriate airway management, and endovascular repair of the injured vessel is critical to the successful management of this potentially life-threatening condition.
